# Potenzielle Auswirkungen der Krankenhausreform und des G-BA-Beschlusses zur Versorgung hüftgelenknaher Femurfrakturen am Beispiel Sachsens: Verbesserung oder Gefährdung der Versorgungsqualität?

**DOI:** 10.1007/s00113-024-01499-x

**Published:** 2024-11-21

**Authors:** Georg Osterhoff, TraumaNetzwerk Westsachsen, TraumaNetzwerk Ostsachsen, Klaus-Dieter Schaser, Christian Kleber

**Affiliations:** 1 Klinik und Poliklinik für Orthopädie, Unfallchirurgie und Plastische Chirurgie, Universitätsklinikum Leipzig, Liebigstr. 20, 04103 Leipzig, Deutschland; 2 UniversitätsCentrum für Orthopädie und Unfallchirurgie, Universitätsklinikum Carl Gustav Carus, Fetscherstr. 74, 01307 Dresden, Deutschland

**Keywords:** Krankenhausreform, Proximale Femurfraktur, Versorgungsqualität, TraumaNetzwerke, Gesundheitspolitik, Orthogeriatrie, Hospital reform, Proximal femoral fractures, Quality of care, Trauma networks, Health policy, Orthogeriatrics

## Abstract

**Hintergrund:**

Die deutschen Kliniken unterliegen aktuellen durch die Krankenhausreform und Richtlinien des Gemeinsamen Bundesausschusses (G-BA) umfangreichen Änderungen.

**Fragestellung:**

Dieser Artikel untersucht die potenziellen Auswirkungen einer *levelbasierten* Krankenhausreform und Umsetzung der G‑BA-Richtlinie zur Versorgung hüftgelenknaher Femurfrakturen in Sachsen.

**Methodik:**

Basierend auf den Eingriffszahlen hüftgelenknaher Femurfrakturen aller zertifizierten Traumazentren in Sachsen (TraumaNetzwerke Ostsachsen und Westsachsen) 2019 und 2022 erfolgte eine Simulation und Visualisierung der Umsetzung der G‑BA-Richtlinie und der Krankenhausreform, um die Auswirkungen auf die notwendigen Kapazitäten und potenziellen Versorgungslücken zu bewerten.

**Ergebnisse:**

Nach Anwendung der Kriterien des G‑BA-Beschlusses zeigt sich eine Reduzierung der hüftgelenknahen Femurfrakturen versorgenden Kliniken in den TraumaNetzwerken Sachsens von 42 auf 28 (−33 %), bei Umsetzung der Krankenhausreform reduziert sich diese Zahl weiter von 42 auf 15 (−64 %). Diese Reduktion würde zu einer erheblichen Steigerung der Fallzahlen in den verbleibenden Kliniken (2- bis 3fach) führen und das bis zu 4Fache in 2030 – mit entsprechend gesteigertem Bedarf an OP-Kapazitäten (1,2 OP/Woche) und ca. 7400 Sekundärverlegungen/Jahr bedeuten. In einzelnen Landkreisen Sachsens stünden nach derzeitigen Maßstäben großflächig keine Klinik zur Versorgung hüftgelenknaher Frakturen zur Verfügung.

**Schlussfolgerung:**

Die geplante Reform und die Umsetzung des G‑BA-Beschlusses bergen nach derzeitigem Stand ein hohes Risiko für Versorgungslücken in Sachsen mit signifikanter Abnahme der an der Versorgung von Patienten mit proximalen Femurfrakturen beteiligten Kliniken und sekundärer Kumulation dieses geriatrischen Patientenguts an den verbleibenden Kliniken. Die notwendigen operativen und stationären Ressourcen in den verbleibenden Kliniken reichen, gemessen am aktuellen Stand, bei Personal- und Kapazitätsmangel nicht zur Versorgung dieser Patienten aus.

**Graphic abstract:**

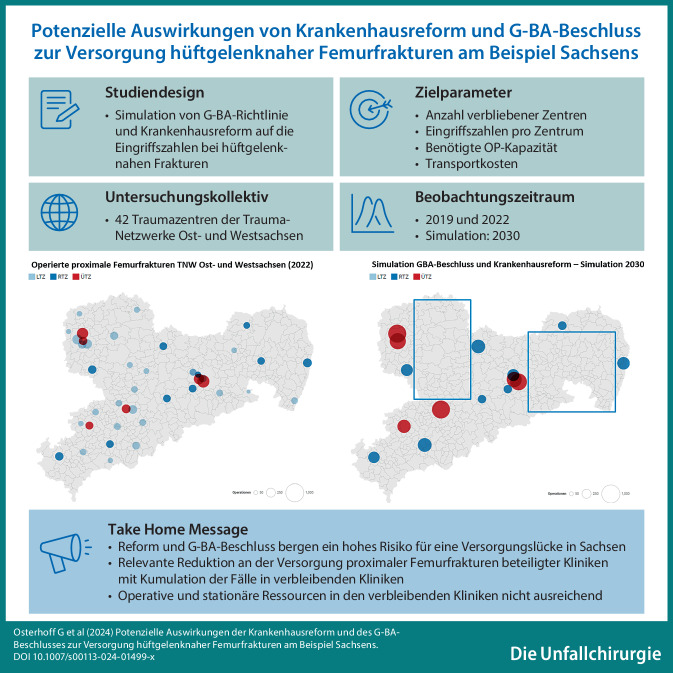

## Abstract (en)

**Background:**

The German hospital landscape is undergoing comprehensive changes due to the increasing aging population and staff shortages in the healthcare sector. These changes are driven by the current hospital reform and the guidelines of the Federal Joint Committee (G-BA) for the treatment of proximal femoral fractures.

**Objective:**

To investigate the effects of the hospital reform and the implementation of the G‑BA guidelines for the treatment of proximal femoral fractures in Saxony.

**Methods:**

Based on the number of proximal femoral fracture surgeries performed in all certified trauma centers in Saxony (East Saxony/West Saxony Trauma Network) in 2019 and 2022, a simulation was conducted to visualize the implementation of the G‑BA guidelines and the hospital reform.

**Results:**

Applying the criteria of the G‑BA resolution results in a reduction of hospitals treating proximal femoral fractures in Saxony from 42 to 28 (−33%). The implementation of the planned hospital reform further reduces the number of such hospitals to 15 (−64%). This reduction leads to a significant increase in case numbers in the remaining hospitals (twofold to threefold) and up to a fourfold increase by 2030. This comes with an increased need for operating capacities (1.2 operating rooms per week) and about 7400 secondary transfers per year. In the districts of North Saxony, Bautzen, Central Saxony, and the Erzgebirge District, no hospital would be available to treat geriatric proximal femoral fractures.

**Conclusion:**

The planned reform and the current implementation of the G‑BA resolution pose a high risk to create gaps in care in Saxony with a markedly reduced number of hospitals involved in treatment of proximal femoral fractures. The necessary operating resources and inpatient capacities in the remaining hospitals are insufficient to care for the expected number of patients given the personnel and capacity shortages.

Die deutsche Krankenhauslandschaft und das Fach Orthopädie/Unfallchirurgie stehen vor weitreichenden Veränderungen. Im Kontext der aktuellen Krankenhausreform sowie der Beschlüsse des Gemeinsamen Bundesausschusses zu hüftgelenknahen Femurfrakturen werden Neuerungen in der Strukturierung und Finanzierung der Krankenhäuser diskutiert und die Verbesserung der Versorgungsqualität angestrebt. In diesem Artikel analysieren wir die potenziellen Auswirkungen dieser Veränderungen im Hinblick auf die Versorgung hüftgelenknaher Femurfrakturen im Bundesland Sachsen.

## Einleitung

Hüftgelenknahe Femurfrakturen sind die zweithäufigsten Frakturen in Deutschland [14]. Aufgrund der zunehmenden Alterung der Bevölkerung ist mit einem Anstieg der Frakturinzidenz in den nächsten Jahrzehnten zu rechnen. In eignen Sächsischen Untersuchungen konnten wir eine 90-Tage-Sterblichkeit von 18 % und für Patienten mit einer Hemiprothese nach medialer Schenkelhalsfraktur nach einem Jahr von 25 %, nach 5 Jahren von 60 % und nach 10 Jahren von 80 % nachweisen [4, 12]. Es handelt sich somit um ein besonders vulnerables Patientenkollektiv. Daher verfolgt der G‑BA-Beschluss zur Versorgung hüftgelenknaher Femurfrakturen laut § 2 das Ziel, eine „Verbesserung der Versorgung dieser Patienten“ zu schaffen. Kern der Richtlinie ist die zeitnahe Versorgung dieser Frakturen binnen 24 h mit interdisziplinärem Management unter Einbindung der Geriatrie. Eine orthogeriatrische Zusammenarbeit konnte in einer Metaanalyse eine Verkürzung der Krankenhausliegedauer, ein 28 % niedrigeres Risiko für Klinikletalität, eine 14 % niedrigere Einjahressterblichkeit und ein 19 % niedrigeres Delirrisiko zeigen [16].

Seit 01.01.2024 sind die hüftgelenknahen Frakturen versorgenden Kliniken dazu verpflichtet, einen Facharzt für Geriatrie frühzeitig einzubinden. Dies kann entweder als eigene Fachabteilung oder durch Kooperation umgesetzt werden, insofern eine tägliche Verfügbarkeit sichergestellt ist.

Am 26.03.2024 wurden in Sachsen erstmals die beiden Universitätskliniken Dresden und Leipzig und nachfolgend weitere Kliniken durch den Medizinischen Dienst der Krankenkassen geprüft. Nach Erstellung des Gutachtens entscheiden nun die Krankenkassen über die Zulassung zur Versorgung hüftgelenknaher Femurfrakturen und damit deren Vergütung. Negative Bescheide an entsprechende Kliniken führen nach Prüfung somit bei gleichbleibender bis eher steigender Inzidenz geriatrischer proximaler Femurfrakturen unmittelbar zur regionalen Umverteilung der Patienten in zugelassene Kliniken zur Versorgung hüftgelenknaher Frakturen und damit Zunahme in Zahl und Länge notwendigen Sekundärverlegungen.

Die Krankenhausreform sieht eine Neustrukturierung der Krankenhausfinanzierung vor, indem sie die Vergütung von Vorhalteleistungen, Krankenhaus-Versorgungsstufen und die Einführung von Leistungsgruppen definiert [1].

Eine unmittelbare Befürchtung ist, dass die gestiegenen Anforderungen und die Umstrukturierung der Krankenhauslandschaft zu Schließungen von Krankenhäusern führen könnten, insbesondere von lokalen Traumazentren in ländlichen und unterversorgten Gebieten [8].

Unabhängig von den Vorgaben des G‑BA-Beschlusses mit Zielsetzung der Verbesserung der Versorgung von Patienten mit proximalen Femurfrakturen und den möglichen Vorgaben der geplanten Krankenhausreform sind jedoch auch die flächendeckende Versorgung sowie die Umsetzbarkeit, insbesondere bezüglich der OP-Kapazität und der Verfügbarkeit an Transportkapazität für die dann notwendigen Sekundärtransporte, bei anstehenden Entscheidungen mitzuberücksichtigen. Die möglichen Folgen für die verbleibenden Krankenhäuser sind vielschichtig. Einerseits könnten sie mit einer erhöhten Patientenzahl konfrontiert werden, da sie die Versorgung der Patienten aus den für die Versorgung dieser Patienten nicht mehr zugelassenen Einrichtungen übernehmen müssten. Andererseits könnte dies auch zu einer Überlastung der vorhandenen Ressourcen führen, insbesondere wenn die verbleibenden Krankenhäuser nicht entsprechend strukturell aufgerüstet und personell verstärkt werden [3]. Um hierfür eine gute Entscheidungsgrundlage zu haben und andererseits die aktuelle Versorgungsrealität darzustellen, haben sich die TraumaNetzwerke Ost- und Westsachsen zusammengeschlossen und eine Analyse für das Bundesland Sachsen durchgeführt.

## Methodik

TraumaNetzwerke sind ein von der Deutschen Gesellschaft für Unfallchirurgie (DGU) initiiertes Element zur Steigerung der Prozess- und Strukturqualität in Kliniken, die auf die Behandlung von Schwerverletzter spezialisiert sind. Diese Netzwerke, bestehend aus lokalen, regionalen und überregionalen Traumazentren sowie den dazugehörigen Rettungsdiensten, verfolgen das Ziel, jedem Schwerverletzten jederzeit eine hochwertige und gleichbleibende Versorgungsqualität zu bieten.

Zugleich sind die TraumaNetzwerke ein Abbild regionaler Strukturen für die Versorgung von Traumapatienten. Hüftgelenknahe Femurfrakturen sind sehr häufige Frakturen, die in allen Versorgungsstufen operativ versorgt werden.

Zur Untersuchung der Folgen der genannten Reformen auf die Traumaversorgung einer Region schien daher die Simulation der Operationszahlen von hüftgelenknahen Femurfrakturen innerhalb eines TraumaNetzwerks geeignet.

Für diese Studie wurden nach Zustimmung aller Leiter der Traumazentren (*n* = 42) in den TraumaNetzwerken Ost- und Westsachsen die Eingriffszahlen bei hüftgelenknahen Femurfrakturen der Jahre 2019 und 2022 in Selbstauskunft erhoben. Die Jahre 2020 und 2021 wurden aufgrund von möglichen Verzerrungen durch die Auswirkungen der COVID-19-Pandemie nicht in die Analyse einbezogen Die Zertifizierungsbescheinigungen für die Lausitzer Seenland Klinikum GmbH – Hoyerswerda als RTZ waren zum Zeitpunkt der Manuskripterstellung noch vorläufig, die Klinik wurde aber in die Analyse einbezogen.

Zusätzlich wurden die Versorgungsstufe (lokales, regionales und überregionales Traumazentrum) und Operation mittels Osteosynthese und Endoprothese erfasst. Die Darstellung der Daten erfolgte mittels Absolut- und Prozentwerten und Gruppenbildung nach Jahr und Versorgungsstufe.

### Simulation des G-BA-Beschlusses

**Abb. 1:**
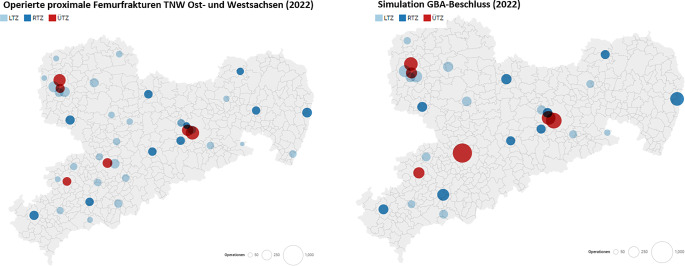
Nach Anwendung der Anforderungen des G‑BA-Beschlusses zeigt sich in Sachsen eine Reduktion der Klinikanzahl zur Versorgung proximaler Femurfrakturen von 42 auf 28

Als erste Stufe wurde die Umsetzung der „Richtlinie zur Versorgung der hüftgelenknahen Femurfraktur“ des G‑BA (Beschluss vom 22.11.2019) simuliert [2]. Dafür wurden bei allen Kliniken die Kriterien geprüft (Stand 10.04.2024) – insbesondere das Vorhandensein einer durchgehenden geriatrisch-konsiliarischen Betreuung. Dies erfolgte durch Recherche auf den Internetauftritten der Kliniken (z. B. Vorhandensein einer geriatrischen Abteilung) *und* Informationen des Landesverbandes Geriatrie Sachsen. *Falls sich aus vorgegangenen Quellen kein Nachweis einer geriatrisch-konsiliarischen Betreuung ergab, erfolgte die telefonische Kontaktaufnahme für eine Selbstauskunft der Kliniken*. Die Eingriffszahlen von 2022 von Kliniken, die die Kriterien des G‑BA-Beschlusses zum 08.04.2024 nicht erfüllten, wurden unabhängig von der Stufe des Traumazentrums auf die verbliebenen Kliniken im Umkreis von 50 km zu gleichen Teilen verteilt (Abb. 1).

Für die verbliebenen Kliniken wurden dann die Fallzahlen berechnet, die 2022 nach Aufteilung der oben erwähnten Fälle an ihrem jeweiligen Traumazentrum versorgt hätten werden müssen.

### Simulation der Krankenhausreform

Als zweite Stufe wurde die Umsetzung der „Dritte[n] Stellungnahme und Empfehlung der Regierungskommission für eine moderne und bedarfsgerechte Krankenhausversorgung – Grundlegende Reform der Krankenhausvergütung“ vom 06.12.2022 simuliert [1]. In diesem ersten Konzeptpapier wird die Einführung sog. Level und nur für bestimmte Level die Vergütung unfallchirurgischer Leistungen empfohlen. Die im Konzeptpapier genannten Kriterien für diese Level decken sich in wesentlichen Punkten mit den Kriterien für die Einstufung als lokales, regionales oder überregionales Traumazentrum. Die in dieser Hinsicht dem Level I entsprechenden lokalen Traumazentren hätten gemäß Konzeptpapier vom 06.12.2022 folglich keine unfallchirurgische Versorgung mehr durchführen dürfen.

Für die Simulation der Krankenhausreform wurden daher die Eingriffszahlen von 2022 der nach Anwendung des G‑BA-Beschlusses verbleibenden lokalen Traumazentren (LTZ) auf die verbleibenden regionalen (RTZ) und überregionalen Traumazentren (ÜTZ) im Umkreis von 50 km zu gleichen Teilen verteilt (Zertifizierung Stand 10.04.2024). Für die verbliebenen Kliniken wurden dann die Fallzahlen berechnet, die 2022 nach Aufteilung der oben erwähnten Fälle versorgt hätten werden müssen.

### Simulation der demografischen Entwicklung

Ausgehend von einer laut Literatur anzunehmenden Zunahme der hüftgelenknahen Femurfrakturen von 2022 bis 2030 um 20 % wurden für die nach Simulation von G‑BA-Beschluss und Krankenhausreform verbliebenen Krankenhäuser die Fallzahlen berechnet, die 2030 wahrscheinlich versorgt werden müssten [14].

### Zuwachs an OP- und Transportkapazitäten

Basierend auf den Fallzahlveränderungen der nach Simulation von G‑BA-Beschluss und Krankenhausreform verbleibenden Krankenhäuser wurde, unter Annahme einer durchschnittlichen Schnitt-Naht-Zeit für die operative Versorgung einer hüftgelenknahen Fraktur von 90 min, der Zuwachs benötigter OP-Kapazitäten ermittelt [7]. Zudem wurde der Zuwachs an benötigten Sekundärverlegungen mitsamt der daraus folgenden Kosten für 2022 und 2030 berechnet. Dabei wurde eine Verlegung in 2022 mit 460,00 € pro Rettungsdiensttransport und in 2030 mit 540 € (angenommene Inflation 2 % p. a.) pro Transport veranschlagt [15].

## Ergebnisse

Insgesamt wurden in den 42 teilnehmenden Krankenhäusern (6 ÜTZ, 10 RTZ, 26 LTZ) der TraumaNetzwerke Ost- und Westsachsen 2019 6617 und 2022 6841 (+3,4 %) hüftgelenknahe Femurfrakturen operativ versorgt (Tab. 1). Dabei versorgten 2019 die LTZ 53,2 % der Frakturen bei einer prozentualen Verteilung an der Gesamtklinikzahl TNW von 61,9 %, 22,8 % für RTZ bei einem Klinikanteil von 23,8 und 24,0 % für ÜTZ bei einem Klinikanteil von 14,3 %. In 2022 kam es zu einer weiteren Abnahme des Versorgungsanteils der proximalen Femurfrakturen in den LTZ (50,6 %) bei Zunahme in den RTZ (24,4 %) und ÜTZ (25 %). In 2022 versorgten 13 von 26 LTZ (50 %), 8 von 10 RTZ (80 %) und alle ÜTZ (100 %) dabei mehr Frakturen als 2019.

**Tab. 1:** Operative Versorgung prox. Femurfrakturen in den Jahren 2019 und 2022 nach Versorgungsstufe

Stufe TNW	Zentren (*n*)	Zentren (%)	Op. 2019	% 2019	Op. 2022	% 2022
LTZ	26	61,9	3518	53,2	3458	50,6
RTZ	10	23,8	1509	22,8	1672	24,4
ÜTZ	6	14,3	1590	24,0	1711	25,0
Gesamt	42	–	6617	–	6841	–

*Op.* Operationen, *LTZ* lokales Traumazentrum, *RTZ* regionales Traumazentrum, *ÜTZ* überregionales Traumazentrum

Die Anwendung der Kriterien des G‑BA-Beschlusses führte in der Simulation zu einer Reduktion von 42 auf 28 Kliniken (−33,3 %), die zur Versorgung hüftgelenknaher Femurfrakturen zugelassen wären (Abb. 1; Tab. 2). In einigen Kliniken führt diese Simulation in 2022 zu mehr als einer Verdopplung allein der Fallzahlen operativ zu versorgender proximaler Femurfrakturen.

**Tab. 2:** Anzahl operativer Versorgungen prox. Femurfrakturen in den einzelnen Traumazentren mit Simulation der Umsetzung von G‑BA-Beschluss (*G‑BA*) und geplanter Krankenhausreform (KHRef)

TZ	Stufe des TNW	2019	2022	2022 G‑BA	2022 G‑BA + KHRef	2030 G‑BA + KHRef
1	LTZ	101	102	0	0	0
2	LTZ	172	165	0	0	0
3	LTZ	91	120	0	0	0
4	LTZ	206	184	195	0	0
5	LTZ	54	67	0	0	0
6	LTZ	255	285	296	0	0
7	LTZ	136	119	0	0	0
8	LTZ	134	121	121	0	0
9	LTZ	284	262	290	0	0
10	LTZ	104	93	0	0	0
11	LTZ	111	98	189	0	0
12	LTZ	86	78	106	0	0
13	LTZ	270	221	0	0	0
14	LTZ	126	132	0	0	0
15	LTZ	64	76	159	0	0
16	LTZ	131	120	157	0	0
17	LTZ	75	74	0	0	0
18	LTZ	130	132	0	0	0
19	LTZ	204	195	212	0	0
20	LTZ	126	130	0	0	0
21	LTZ	55	39	79	0	0
22	LTZ	146	168	0	0	0
23	LTZ	105	116	0	0	0
24	LTZ	139	146	175	0	0
25	LTZ	127	129	129	0	0
26	LTZ	86	86	126	0	0
27	RTZ	146	169	217	462	554
28	RTZ	163	165	292	472	566
29	RTZ	156	201	201	322	386
30	RTZ	109	100	185	352	423
31	RTZ	150	166	166	166	199
32	RTZ	181	158	0	0	0
33	RTZ	131	166	166	166	199
34	RTZ	98	123	163	176	211
35	RTZ	200	224	394	386	463
36	RTZ	175	200	200	349	418
37	ÜTZ	211	195	254	656	787
38	ÜTZ	163	177	258	437	524
39	ÜTZ	291	362	390	823	988
40	ÜTZ	225	224	800	816	979
41	ÜTZ	293	313	398	565	679
42	ÜTZ	407	440	525	692	831
*Gesamt*		*6617*	*6841*	*6841*	*6841*	*8209*

*LTZ* lokales Traumazentrum, *RTZ* regionales Traumazentrum, *ÜTZ* überregionales Traumazentrum

**Abb. 2:**
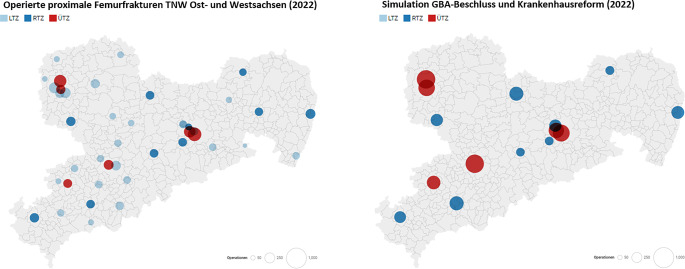
Nach Anwendung der Anforderungen des G‑BA-Beschlusses und der Krankenhausreform zeigt sich in Sachsen eine Reduktion der Klinikzahl zur Versorgung prox. Femurfrakturen von 42 auf 15

**Abb. 3:**
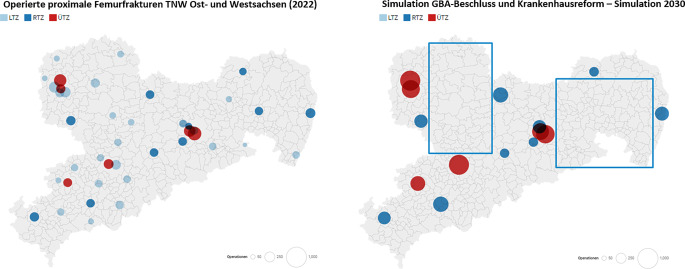
Simulation für das Jahr 2030 (*rechts*) nach Anwendung der Anforderungen des G‑BA-Beschlusses und der Krankenhausreform und unter Annahme einer Zunahme der hüftgelenknahen Femurfrakturen um 20 % bis 2030 [14]

Die Simulation der Krankenhausreform gemäß den Kriterien des Konzeptpapiers vom 06.12.2022 führte zu einer weiteren Reduktion auf 15 der ursprünglich 42 Kliniken (−64,3 %), welche proximale Femurfrakturen versorgen dürften (Abb. 2). Das würde in einigen verbleibenden Kliniken zu einer Verdreifachung der Fallzahlen operativ zu versorgender proximaler Femurfrakturen im Jahr 2022 und zu einer Vervierfachung der Fallzahlen bis 2030 führen (Abb. 3 und 4).

**Abb. 4:**
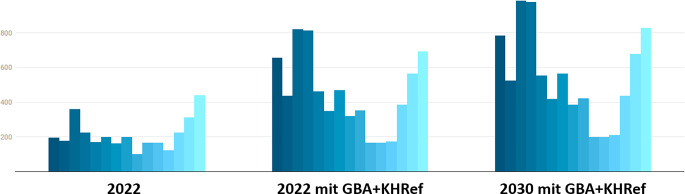
Prognostizierte Zunahme der Fallzahlen hüftgelenknaher Femurfrakturen für die verbleibenden Krankenhäuser nach Anwendung der Anforderungen des G‑BA-Beschlusses und der Krankenhausreform (KHRef). *Balken gleicher Farbe* entsprechen jeweils einem Krankenhaus in Sachsen

Für alle verbleibenden Kliniken würde die Anwendung beider Regularien und bei einer angenommenen durchschnittlichen Operationsdauer von 90 min [7] zu einem zusätzlichen Bedarf an OP-Kapazität von 5424 h oder 678 OP à 8 h in 2022 und von 7436 h oder 930 OP à 8 h in 2030 führen.

Das entspräche 1,2 OP pro Woche je verbleibender Klinik in 2030 allein für die Versorgung zusätzlich anfallender hüftgelenknaher Femurfrakturen.

Die erforderlichen Sekundärverlegungen aus Kliniken, die keine hüftgelenknahen Femurfrakturen versorgen dürften, in die verbleibenden Zentren würden sich in 2022 auf 3616 summieren und zusätzliche Kosten von 1.663.360 € verursachen bzw. 4957 und 2.675.766 € in 2030 (unter Annahme von 2 % Inflation p. a.).

Geht man davon aus, dass diese Patienten – wie in Planungen zur Krankenhausreform angedacht – mangels Bettenkapazitäten auch heimatnah rückverlegt werden müssen, würden sich die Verlegungen entsprechend verdoppeln (2022: 7232/3.326.720 €, 2030: 9914/5.351.532 €).

## Diskussion

Eigene Studien zur Versorgung von proximalen Femurfrakturen in Sachsen mit hoher Mortalität unterstreichen das besonders schützenswerte Kollektiv von Patienten mit hüftgelenknahen Frakturen [4, 12]. Die dem G‑BA-Beschluss zugrunde liegende besondere Zuwendung mit unbedingt erforderlichem geriatrischem Screening und interdisziplinärer Behandlung zur Senkung der perioperativen Morbidität und Mortalität ist daher sinnvoll, und die unbedingte Umsetzung dieser Beschlüsse steht nach einheitlicher Auffassung aller an der Behandlung proximaler Femurfrakturen beteiligten Disziplinen absolut nicht zur Diskussion. Der Weg zur interdisziplinären Absicherung und schrittweisen Etablierung der geforderten Struktur- und Prozessanforderungen als Grundvoraussetzung zur flächendeckenden Umsetzung der Beschlüsse ist jedoch aus Sicht der Autoren diskutabel.

Die Definition von notwendigen Rahmenbedingungen wie Notaufnahme, Fachabteilungen und Intensivstation ist aus Sicht der Autoren eine gut geeignete Maßnahme zur Standardisierung einer Versorgung anlog dem Vorbild der TraumaNetzwerke zur Schwerverletztenversorgung.

Die zeitdringliche operative Versorgung binnen 24 h sollte hingegen individuell betrachtet werden. In eigenen sächsischen Studien konnten wir aktuell zeigen, dass das Überleben signifikant von den Komorbiditäten (Charlson Comorbidity Index) und nicht von Operationszeitpunkt und Operateur abhängig ist [4, 12]. Patienten, welche aufgrund der Dekompensation einer internistischen Erkrankungen oder akuten Infektion stürzen, profitieren wahrscheinlich von einer präoperativen Konditionierung, was Gegenstand weiterer Untersuchung sein sollte.

Die tägliche Verfügbarkeit einer Physiotherapie auch am Wochenende ist aus Sicht der Autoren entscheidend zur Umsetzung der schnellen Mobilisation, wenngleich dies einzelne Klinken vor enorme personelle Herausforderungen stellt. In ländlichen Bereichen Sachsens herrscht ein eklatanter Mangel an Physiotherapeuten. Eine physiotherapeutische Anstellung in einer Klinik mit Wochenenddiensten erscheint hierbei wenig attraktiv und kann nur schwierig in der Personaleinsatzplanung abgesichert werden.

Zudem haben sich die Rahmenbedingungen seit dem G‑BA-Beschluss 2019 nach der Coronapandemie grundlegend geändert. Das aktuelle Gesundheitssystem ist geprägt durch gerade in der Kranken- und OP-Pflege bestehenden enormen Personalmangel, dessen Spürbarkeit sich in den einzelnen Kliniken durch die Einführung von Pflegepersonaluntergrenzen noch weiter verschärft hat [5]. Dieser Personalmangel führt in den operativen Fächern zu einer reduzierten Nutzbarkeit von OP-Kapazitäten. Zudem herrscht ein enormer ökonomischer Druck, weshalb einzelne Kliniken sich mit Blick auf eine Erlösoptimierung auf die Versorgung DRG-lukrativer Fälle und Operationen fokussieren. Hierbei scheinen zeitkritisch – und damit auch außerhalb der Regelarbeitszeit – zu versorgende hüftgelenknahe Frakturen der größtenteils polymorbiden geriatrischen Patienten aufgrund des hohen perioperativen Riskos und Pflegaufwands bei gleichzeitig überschaubarem ökonomischen Anreiz durch die vom G‑BA-Beschluss getriggerten steigenden Anforderungen weniger interessant geworden zu sein. Somit besteht aus Sicht der Autoren das Risiko, dass sich Kliniken aus der Versorgung hüftgelenknaher Frakturen zurückziehen und sich zumindest anteilig auf die Durchführung geplanter Operation und elektiver Eingriffe konzentrieren. In den verbleibenden Kliniken kommt es konsekutiv zu einer Kumulation dieses polymorbiden Patientenguts mit einer Verschiebung des in diesen Kliniken versorgten Patientenspektrums hin zu geriatrischer Notfallversorgung. Aktuell würde dafür nicht nur ausreichend freie OP-Kapazität zur Versorgung der vornehmlich geriatrischen Patienten mit proximalen Femurfrakturen fehlen, sondern die enorme Verknappung von operativen, intensivmedizinischen und stationären Ressourcen hätte eine deutlich reduzierte Versorgbarkeit anderer, auch geriatrischer Notfallpatienten (Becken‑/Wirbelsäulenpatienten, die in Traumazentren niedriger Stufe ohnehin nicht behandelbar wären), aber auch elektiver muskuloskeletaler/onkologischer Eingriffe zur Folge.

Die Simulation der Auswirkungen des G‑BA-Beschlusses und der Krankenhausreform auf die Versorgung hüftgelenknaher Frakturen in Sachsen zeigt eine drastische Reduzierung der Anzahl der Krankenhäuser in Sachsen von 42 auf letztlich 15 (−64,3 %). Diese Reduktion führt zu einer erheblichen asymmetrischen Steigerung der Fallzahlen in den verbleibenden Kliniken und einem entsprechend gestiegenen Bedarf an Operationskapazitäten und Sekundärverlegungen, was wiederum deutlich höhere Kosten verursacht. In einigen Landkreisen Sachsens stünde großflächig keine Klinik entsprechend den Anforderungen zur Versorgung hüftgelenknaher Frakturen zur Verfügung.

Seit 2019 findet ohnehin schon eine Umverteilung der Patienten mit hüftgelenknahen Frakturen von einzelnen lokalen Traumazentren auf regionale und überregionale Traumazentren statt. Dies führt schon jetzt in überregionalen Traumazentren zu einem Versorgungskapazitätsmangel. Die Umsetzung des G‑BA-Beschlusses würde dies weiter aggravieren. Zeitdringliche Versorgungen der Patienten mit hüftgelenknahen Frakturen kollidieren mit der Versorgung anderer komplexer Fälle wie Schwerverletzte, Verletzungen nach dem Verletzen (VAV)- und Schwerverletztenartenverfahren (SAV) der gesetzlichen Unfallversicherungen. Zudem werden diese Kliniken aus Sicht der Autoren zunehmend unattraktiv für das medizinsuche Personal, da eine Arbeitsverdichtung und hohes Notfallaufkommen mit nächtlichen Operationen und entsprechender Belastung resultiert. Diese Entwicklung in Verbindung mit steigenden Fallzahlen veranlasst in der aktuellen Erfahrung noch mehr Pflegekräfte, ihre Arbeitszeit zu verkürzen oder aus dem Beruf auszusteigen [5].

Als methodische Limitation der vorliegenden Analyse sei genannt, dass es in Sachsen 9 Kliniken gibt, die proximale Femurfrakturen versorgen und nicht in einem der beiden analysierten TraumaNetzwerke Ost- und Westsachsen organisiert sind. Die Stichprobe repräsentiert somit 82 % aller proximale Femurfraktur versorgenden Kliniken in Sachsen. Alle Maximalversorger und alle Traumazentren nach KHEntG in Sachsen sind in der Analyse enthalten, und die Verlegungspfade in den TNW sind entsprechend den Netzwerkvereinbarungen geregelt. Zusätzliche, bisher nicht in die Analyse aufgenommene grund- und regelversorgende Krankenhäuser würden der Grundaussage der Studie nicht widersprechen, sondern den beschriebenen Effekt einer Kumulation von Patienten mit hüftgelenknahen Femurfrakturen in den größeren Häusern noch verstärken.

Aus Sicht der TraumaNetzwerke in Sachsen sind eine Umsetzung und die damit verbundene Umverteilung der Patienten in Kliniken einer höheren Versorgungsstufe ohne Zuführung von Personal- und OP-Kapazität nicht möglich.

Die Ergebnisse unserer Studie spiegeln die diskutierten Bedenken bezüglich der Konsequenzen von Krankenhausreformen wider [3, 6, 8–10]. Studien, die sich mit den Auswirkungen von Krankenhausschließungen auf die Patientenversorgung befassen, bestätigen häufig eine Zunahme der Belastung verbleibender Einrichtungen und eine potenzielle Gefährdung der Patientenversorgung, insbesondere in ländlichen Gebieten [3, 8–10]. Diese Ergebnisse stützen daher die Hypothese, dass eine solche Reform, ohne angemessene Planung und Ressourcenallokation, zu einer Verschlechterung der Versorgungsqualität führen könnte. Zudem bleiben wesentliche Aspekte wie z. B. die notwendige Zahl an Geriatern und die Transportkapazität unberücksichtigt. In einer durch die Autoren angeforderten Stellungnahme des Landesverbandes für Geriatrie in Sachsen wird diese Problematik ebenfalls aufgegriffen. 47 % der unfallchirurgischen Kliniken mit nachgewiesener Versorgung von hüftgelenknahen Frakturen würden eine externe geriatrische Konsilversorgung benötigen.

Eine aktuelle Abbildung des Berufsverbandes Geriatrie (https://www.bv-geriatrie.de/, abgerufen am 28.05.2024) zeigt deutliche Lücken der stationären geriatrischen Versorgung in Sachsen. Diese decken sich mit unserer Simulation der Versorgung hüftgelenknaher Frakturen in Sachsen. Hierbei sind v. a. die Landkreise Nordsachsen, Mittelsachsen, Bautzen und der Erzgebirgskreis von entsprechenden geriatrischen Versorgungsengpässen betroffen.

Unsere Studie basiert auf Controlling-basierten Selbstauskünften der Kliniken in den sächsischen TraumaNetzwerken sowie auf Simulationen und Annahmen, die trotz sorgfältiger Ausarbeitung und Anlehnung an aktuelle Daten und Richtlinien gewisse Unsicherheiten bergen. Bei der Abfrage der Versorgungszahlen von Patienten mit proximaler Femurfraktur aller in den TraumaNetzwerken Ost- und Westsachsen organisierten Kliniken wurden alle operierten Patienten erfasst. Von einem positiven geriatrischen Assessment und damit geriatrischer Mitbehandlung ist ungefähr bei 85–90 % aller Patienten auszugehen, sodass die dargestellten Zahlen leicht zu hoch sein könnten. Andererseits sind nur ca. 90 % aller an der proximalen Femurfrakturversorgung beteiligten sächsischen Kliniken in TraumaNetzwerken organisiert. Diese Versorgungszahlen sind in unseren Abfragen nicht erfasst und würden bei entsprechender Berücksichtigung wiederum zu einer Steigerung der dargestellten Eingriffszahlen führen. Daher bedarf es eines dringenden Abgleichs dieser aus den TraumaNetzwerken stammenden Eingriffszahlen mit den entsprechenden Abrechnungsdaten der Krankenkassen mit spezifischer Ausweisung der Fallzahlen mit verpflichtender geriatrischer Mitbehandlung.

Auch wurde in der Simulation von einer gleichteiligen Verteilung des zusätzlichen Patientenaufkommens nach reformbedingtem Wegfall eines Krankenhauses auf die nächstliegenden benachbarten Kliniken mit entsprechender Zulassung zur Versorgung dieser Patienten ausgegangen. Die realen Auswirkungen können durch regionale Besonderheiten, unvorhersehbare Entwicklungen im Gesundheitssektor oder politische Veränderungen beeinflusst werden und im Einzelfall dazu führen, dass die zusätzliche Belastung für einige Häuser noch drastischer als in dieser Simulation ausfällt. Hinsichtlich der Krankenhausreform wurde in der politischen Diskussion die initiale Idee von Versorgungsstufen bereits wieder weitgehend verlassen, sodass der Effekt weniger stark sein dürfte. Da die Diskussion jedoch noch nicht abgeschlossenen ist, erachten die Autoren die Betrachtung evtl. Effekte auch für zukünftige politische Entscheidung für wichtig und wertvoll. Das gewollte Ziel der Reform ist eine Reduktion der Zahl kleiner Krankenhäuser in der Peripherie mit einer Zentralisierung der Krankenhauslandschaft ist.

Auch die Einführung von rein elektiv-chirurgischen Leistungsgruppen wird dazu führen, dass viele Krankenhäuser wirtschaftlich nicht mehr überleben werden können – und dann auch keine proximalen Femurfrakturen mehr versorgen. Die exakte Ausgestaltung der Reform in den anderen Bundesländern ist bisher vollkommen ungewiss und muss auch den Besonderheiten der einzelnen Länder Rechnung tragen (z. B. Metropolregion vs. Flächenland). Ziel dieser Analyse war es aber auch, explizit „Worst-Case-Szenarios“ aufzuzeigen, welche im Sinne der Versorgungssicherheit und -qualität unserer Patienten vermieden werden müssen.

Zudem wurden weitere Faktoren wie die Auswirkungen auf das Personal nicht berücksichtigt. Der in den für die proximale Femurfrakturversorgung zugelassenen Traumazentren infolge stark vermehrten Aufkommens geriatrischer Patienten erhöhte Personalbedarf ist ebenfalls nicht berücksichtigt. Ferner ist die Zunahme an Krankenstand in Phasen von und an Einrichtungen mit erhöhter Arbeitsbelastung in der Literatur belegt und wäre hier als weiterer die Versorgungssituation verschärfender Umstand zu berücksichtigen [11, 13]. Zukünftige Studien sollten sich auf die langfristigen Auswirkungen dieser Reformen auf die Ergebnisqualität der Patientenversorgung, insbesondere in unterversorgten und ländlichen Gebieten, konzentrieren. Eine detaillierte Untersuchung der Auswirkungen auf die Mitarbeiterzufriedenheit und -belastung sowie auf Patientensicherheit und -outcome könnte weitere wichtige Erkenntnisse liefern. Zudem wäre eine Evaluation der ökonomischen Folgen dieser Reformen auf das Gesundheitssystem, insbesondere auf die Verweildauern, den erhöhten Personalaufwand unfallchirurgischer wie auch akutgeriatrischer Krankenhausabteilungen und deren Finanzierbarkeit in den gegenwärtigen Erlössystemen insgesamt von großem Interesse.

## Fazit für die Praxis

- Die geplante Reform und Umsetzung des G‑BA-Beschlusses birgt ein hohes Risiko zur Verschlechterung der Versorgungsqualität hüftgelenksnaher Femurfrakturen und potenziellen Versorgungslücken in Sachsen.
- Keine Berücksichtigung der Sekundärverlegungen im Rettungsdienst
- Keine ausreichenden Ressourcen für die operative, intensivmedizinische und stationäre Versorgung an den verbleibenden Kliniken vorhanden. Aus unserer Sicht sollten nachfolgende Aspekte berücksichtigt werden:
  - Sicherung der Versorgung von hüftgelenknahen Frakturen in der Fläche mit Vermeidung langer Transportwege und der Kumulation dieser Patienten in wenigen größeren Traumazentren
  - Planung auf Landesebene unter Berücksichtigung der Transportkapazität
  - Dauerhafte Akzeptanz der Telemedizin als Möglichkeit für Kliniken ohne stationäre Fachabteilung für Geriatrie zur Sicherstellung der orthogeriatrischen G‑BA-Vorgaben
  - Konsequenter Ausbau der geriatrischen Versorgungsstrukturen
  - Anpassung der DRG zur Refinanzierung des Mehraufwandes

## Data Availability

Die erhobenen Datensätze können auf begründete Anfrage in anonymisierter Form beim korrespondierenden Autor angefordert werden. Die Daten befinden sich auf einem Datenspeicher am Universitätsklinikum Leipzig.
